# Isolated thumb carpometacarpal joint dislocation: a case report and review of the literature

**DOI:** 10.1186/1749-799X-5-16

**Published:** 2010-03-10

**Authors:** Elias Fotiadis, Theodoros Svarnas, Christos Lyrtzis, Alexis Papadopoulos, Panagiotis Akritopoulos, Byron Chalidis

**Affiliations:** 1 Orthopaedic Department, General Hospital of Veria, (Verias-Asomaton), Veria, (59100), Greece; 2 1st Orthopaedic Department, Aristotle University of Thessaloniki, (Ag. Dimitriou), Thessaloniki, (54622), Greece; 3 Orthopaedic Department, Avenue Hospital, Melbourne, Australia

## Abstract

**Background:**

Isolated thumb carpometacarpal dislocation is a rare injury pattern and the optimal treatment option is still controversial.

**Case Description:**

We present a 27-year-old basketball player who underwent an isolated dorsal dislocation of the thumb carpometacarpal joint after a fall. The dislocation was successfully reduced by closed means but the joint was found to be grossly unstable. Due to inherent instability, repair of the ruptured dorsoradial ligament and joint capsule was performed.

The ligament was detached from its proximal insertion into trapezium and subsequently stabilized via suture anchors. The torn capsule was repaired in an end-to-end fashion and immobilization of the joint was applied for 6 weeks.

**Results:**

At 3-year follow up evaluation the patient was pain free and returned to his previous level of activity. No restriction of carpometacrpal movements or residual instability was noticed. Radiographic examination showed normal joint alignment and no signs of subluxation or early osteoarthritis.

**Conclusion:**

Surgical stabilization of the dorsal capsuloligamentous complex may be considered the selected treatment option in isolated carpometacarpal joint dislocations, that remain unstable after closed reduction in young and high demand patients.

**Level of Clinical Evidence: **Level IV

## Introduction

Isolated dislocation of the carpometacarpal (CMC) joint of the thumb is an uncommon upper limb and hand injury. The lesion is usually the consequence of an axial transmitted force through a partially flexed thumb. Due to thick and strong volar ligamentous complex the dislocation occurs in dorsal direction through the thin dorsal capsule [1,2].

The optimal treatment strategy for the acute thumb CMC joint dislocation remains a subject of debate. Closed reduction and casting, closed or open reduction along with transfixion with Kirschner wires and reconstruction of dorsal ligament and capsuloraphy have been performed so far according to joint stability and surgeon's preference. However, only few cases have been reported in the literature and a universally accepted protocol has not been developed yet [3].

We report a case with an acute isolated thumb carpometacarpal dislocation that was treated with reconstruction of the dorsal capsuloligamentous structures. The three-year follow up outcome, as well as review of the literature for similar cases are presented.

This study was approved by the scientific review board at our hospital and was conducted in accordance with the World Medical Association Declaration of Helsinki of 1964, as revised in 1983. **Written informed consent was obtained from the patient for publication of this case report and accompanying images**.

## Case Description

**Figure 1:**
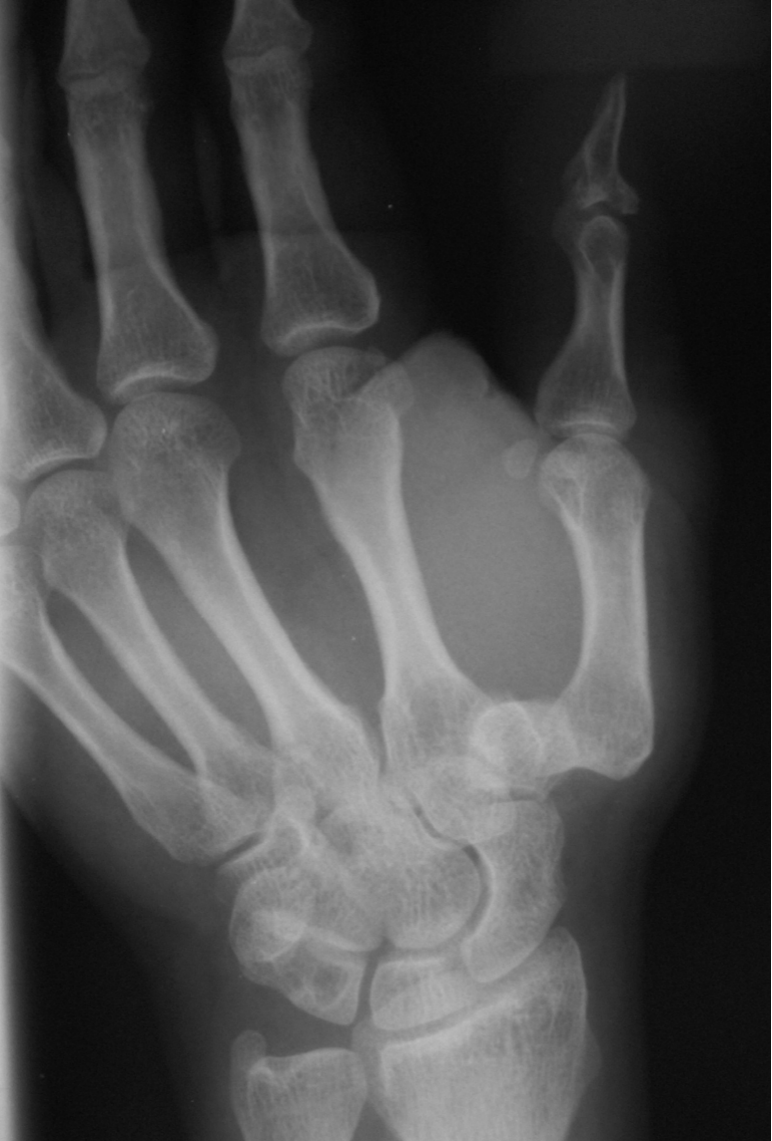
**Anteroposterior radiograph of the right hand**. Isolated thumb CMC joint dislocation is evident.

A 27-year-old, right-hand dominant basketball player was admitted on emergency department of our hospital, after a fall during a basketball game and injury of the right hand. Clinical examination revealed a deformity and swelling at the dorsoradial side of the hand in association with tenderness and pain in thumb movements. Anteroposterior and oblique hand and thumb radiographs demonstrated an isolated dorsal dislocation of the CMC joint. No fracture signs were identified (Figure 1).

**Figure 2:**
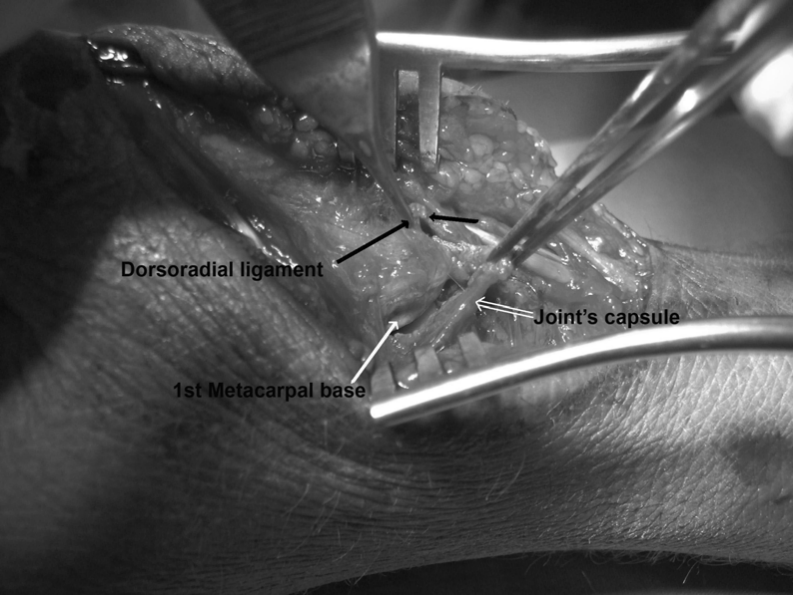
**Intraoperative photograph of the dorsal aspect of carpometacarpal joint**. The dorsoradial ligament (black arrow) has been detached from its attachment to trapezium. The capsule (double white arrow) has been also transversely torn exposing the joint and the base of 1^st ^metacarpal (white arrow).

Intra-articular injection of local anaesthetic (xylocaine 2%) was followed by closed reduction of the carpometacarpal joint dislocation. However, the joint found to be grossly unstable and reconstruction of the dorsal capsuloligamentous complex occurred. The procedure was performed within few hours of the injury under regional anaesthesia using a dorsoradial approach. The dorsoradial ligament of CMC joint was found to be completely torn from its proximal insertion leaving a small cuff attached on the trapezium. The joint capsule was also transversely torn in its mid-substance but no articular cartilage lesions in both joint sides were evident. (Figure 2). The volar ligament was remained also intact. After debridement of the dorsal surface of the trapezium the dorsoradial ligament was stabilized onto trapezium using a Mini-Mitec suture anchor loaded with a 2-0 suture material (Ethibond). Furthermore, the CMC joint capsule was repaired in an end-to-end fashion with 3-0 Vicryl interrupted stitches. Wound closure was followed by application of a short-arm spica cast for approximately 6 weeks. Afterwards, active and passive movements in the joint were commenced but any hand-played sports were prohibited for another 6 weeks.

## Results

**Figure 3:**
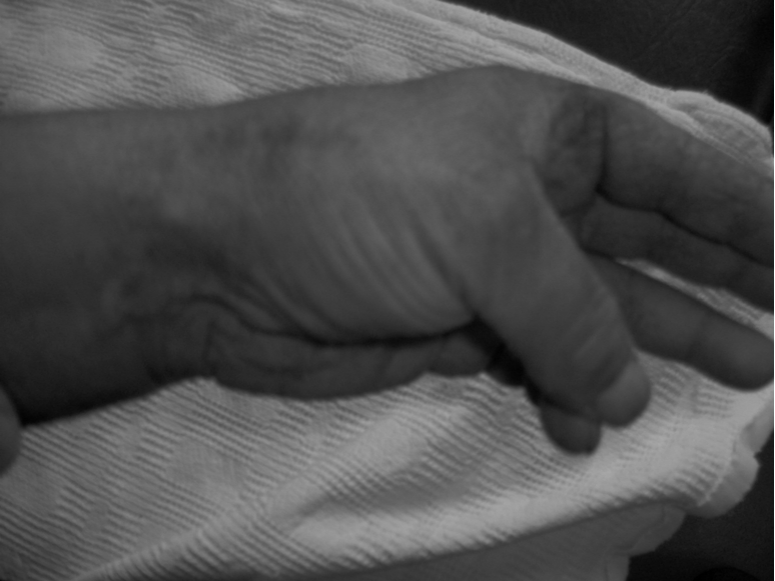
**Appearance of the right hand 3 years post-operatively**. The patient had normal and painless thumb movement.

**Figure 4:**
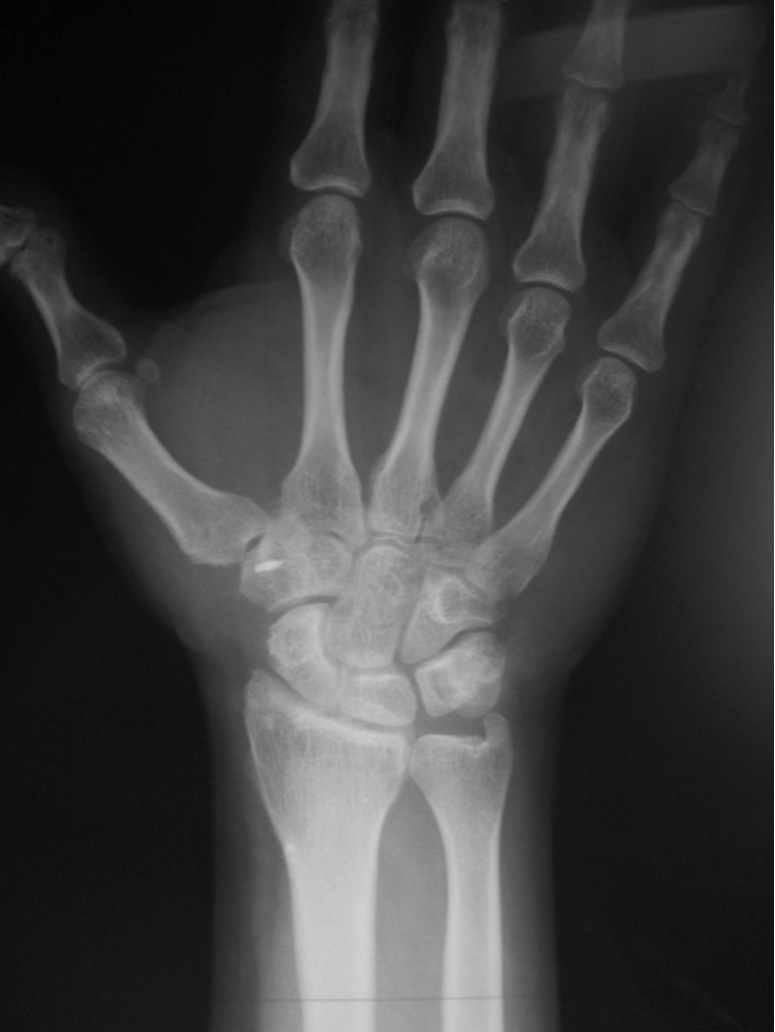
**Anteroposterior radiograph of the right hand 3 years post-operatively**. Good joint congruency without signs of instability or osteoarthritis are seen.

At 3-year follow-up, the patient was pain free and returned to the pre-injury level of activity. No limitation of thumb carpometacarpal joint mobility or residual instability was observed (Figure 3). Radiographic examination revealed normal joint anatomy without any signs of subluxation or early osteoarthritis (Figure 4).

## Discussion

The curved articular surfaces of CMC joint provide only limited stability, compared to the ligaments embedded within the joint capsule. Ligaments do not only represent the primary source of joint stability, but also set the limits of motion in conjunction with the passive tension of muscles [4]. Therefore, their integrity is essential to maintain the static and dynamic stability between the 1^st ^metacarpal bone and trapezium. Excessive laxity of the ligaments, such as after CMC dislocation, may lead to joint instability and subsequently to degeneration of the articular cartilage [4].

Isolated CMC dislocation is associated with various degrees of joint capsule and ligament damage. The volar or anterior oblique ligament is a short and strong structure that was considered for many years the basic key stabilizer for preventing dorsal dislocation of the joint [1]. Bettinger et al. [5] were further reported that the anterior oblique as well as the radial collateral and the ulnar collateral ligaments should be considered the main dynamic stabilizers of the thumb.

However, Strauch et al [6] in a cadaveric found that the dorsoradial ligament complex was the primary restraint to dorsal dislocation and responsible for obtaining joint stability in thumb opposition. Moreover, the authors found that it could be also responsible for joint stability in thumb opposition. This finding was confirmed clinically from Shah and Patel [7] who noticed no disruption of volar capsule or ligament in 4 cases with thumb CMC dislocation. Conversely, the dorsal capsule and ligament found to be avulsed or torn. In our patient, we similarly observed that the dorsal capsuloligamentous complex was completely ruptured but the integrity of volar ligament was well preserved.

The healing potential of dorsal elements without any surgical intervention is still a controversial issue and the indications for performing early ligament reconstruction have not been clearly defined. Conservative or minimally invasive methods (percutaneous pinning) have been applied by some authors. (Table 1) Watt and Hooper [8] described the result of closed reduction and cast or cast and K-wire fixation in 12 patients. One third of patients who treated with cast only and two thirds of patients who treated with cast and K-wire fixation had an unstable and dorsally subluxating joint, which caused weakness and discomfort on hand gripping. Jacobsen and Elberg [3] reported a case with isolated thumb CMC dislocation that was treated with closed reduction and K-wire fixation. Eighteen months post-injury, slight instability and radial subluxation of the first metacarpal bone was found. In the latter scenario, ligament reconstruction by using the Eaton and Littler technique can be applied. The operation has offered good functional results and adequate pain relief in patients with chronic CMC instability after traumatic dislocation of the thumb [9].

**Table 1:** Published cases with isolated thumb CMC dislocation in English literature

Study	Year	Number of cases	Treatment	Result
**Shah J and Patel^7^** *Clin Orthop Relat Res*	1983	4	**A**. Open reduction + pinning (2 patients) **B**. Closed reduction pinning (1 patient) **C**. Open reduction + cast in (1 patient)	**A**. Dorsal subluxation, mild arthritic changes. **B **and **C**. No subluxation - Normal range of motion

**Watt N and Hooper G**^8^ *J Hand Surg*	1987	12	**A**. Closed reduction + cast (6 patients) **B**. Closed reduction + cast after 3-21 days (3 patients) **C**. Closed reduction + pinning + cast (3 patients)	**A**. Asymptomatic instability **B**. Pain and instability **C**. No pain or instability

**Chen VT**^2^ *J Hand Surg (Br)*	1987	1	Ligament reconstruction	Good functional result

**Jacobsen CW and Elberg JJ**^3^ *Scand J Plast Reconstr Surg Hand Surg*	1988	1	Closed reduction + pinning	Slight instability

**Simonian PT and Trumble TE**^12^ *J Hand Surg (Am)*	1996	17	**A**. Closed reduction + pinning (8 patients) **B**. Early ligamentous reconstruction (9 patients)	**A**. Revision surgery for recurrent instability in 4 patients (50%) **B**. Normal grip strength and range of motion

**Kural C et al**^11^ *Acta Orthop Traum Turc*	2002	1	Closed reduction + cast	No pain or instability

**Khan AM et al**^10^ *Am J Orthop*	2003	1	Closed reduction + cast	Good functional result

**Bosmans et al**. ^1^ *J Hand Surg (Am)*	2008	2	Closed reduction + cast	No instability-Normal range of motion

On the other hand, Bosmans et al [1] obtained good result in 2 patients with isolated thumb CMC joint dislocation after closed reduction and cast. Three year post-injury the patients were pain free and had normal range of motion. Similarly, a very satisfactory outcome was noticed by Khan et al [10] in another patient with bilateral thumb CMC dislocation that treated with closed reduction and cast. Kural et al [11] achieved also good result after closed reduction and cast of a unilateral thumb CMC dislocation.

Simonian and Trumble [12] compared early ligamentous reconstruction with closed reduction and pinning. Four out of 8 patients who initially treated with closed reduction and percutaneous pinning showed recurrent instability. In reconstructive group (minimum follow-up period of 2 years), painless full range of motion and normal grip strength were observed. A good result was seen also from Chen VT [2] in a patient who treated with dorsal ligament reconstruction. Shah and Patel [7] advocated that open reduction and K-wire fixation without ligament reconstruction might not be adequate for this type of injury. In their series 2 patients had dorsal subluxation of thumb metacarpal bone after application of the above technique. The remaining patients who underwent open reduction and K-wire fixation or closed reduction and percutaneous pinning respectively had congruent joints.

The above cases point out the unpredictable outcome of conservative or minimally operative treatment modalities in stability of thumb CMC joint. Inadequate treatment may increase the incidence of recurrent instability, joint degeneration and chronic pain and negatively affect the long-term functional result[11]. Bosmans et al [1] suggested that a nonoperative protocol should be followed in case of joint congruency after successful closed reduction. The authors mentioned that ligament reconstruction was not clearly justified and should be avoided in acute cases. However, it seems that closed or open reduction and percutaneous pinning can not always guarantee an optimum result and ligament reconstruction should not be considered a superfluous treatment option.

## Conclusion

We believe that an unstable thumb CMC joint after closed reduction of dorsal dislocation probably illustrates a more serious damage in dorsal ligament and joint capsule. In this case, dorsal capsuloraphy and ligament repair may be of clear benefit particularly in young athletes with high upper extremity demand.

## Competing interests

The authors declare that they have no competing interests.

## Authors' contributions

E. F. was a major contributor in writing the manuscript while B. C. was a major contributor in writing and in editing the manuscript, as well. C. L. and T.S. analyzed and interpreted the patient data regarding the injury. A. P. and P.A. have been involved in drafting the manuscript.
